# Metabolism of waste engine oil by *Pseudomonas* species

**DOI:** 10.1007/s13205-016-0419-5

**Published:** 2016-04-08

**Authors:** Lateef B. Salam

**Affiliations:** Department of Biological Science, Microbiology Unit, College of Natural Sciences, Al-Hikmah University, Ilorin, Kwara Nigeria

**Keywords:** Biodegradation, *Pseudomonas aeruginosa*, Waste engine oil, Pristane, Phytane

## Abstract

Two bacterial strains phylogenetically identified as *Pseudomonas aeruginosa* strains RM1 and SK1 displayed extensive degradation ability on waste engine oil (SAE 40W) in batch cultures. Spectrophotometric analysis revealed the presence of various heavy metals such as lead, chromium and nickel in the waste engine oil. The rate of degradation of waste engine oil by the isolates, for the first 12 days and the last 9 days were 66.3, 31.6 mg l^−1^ day^−1^  and 69.6, 40.0 mg l^−1^ day^−1^ for strains RM1 and SK1, respectively. Gas chromatographic (GC) analyses of residual waste engine oil, revealed that 66.58, 89.06 % and 63.40, 90.75 % of the initial concentration of the waste engine oil were degraded by strains RM1 and SK1 within 12 and 21 days. GC fingerprints of the waste engine oil after 12 days of incubation of strains RM1 and SK1 showed total disappearance of C_15_, C_23_, C_24_, C_25_ and C_26_ hydrocarbon fractions as well as drastic reductions of C_13_, C_14_, C_16_ and PAHs fractions such as C_19_-anthracene and C_22_-pyrene. At the end of 21 days incubation, total disappearance of C_17_-pristane, C_22_-pyrene, one of the C_19_-anthracene and significant reduction of C_18_-phytane (97.2 %, strain RM1; 95.1 %, strain SK1) fractions were observed. In addition, <10 % of Day 0 values of medium fraction ranges C_13_, and C_16_ were discernible after 21 days. This study has established the potentials of *P. aeruginosa* strains RM1 and SK1 in the degradation of aliphatic, aromatic and branched alkane components of waste engine oils.

## Introduction

Waste engine oil is a brown to black oil removed from automobiles when oil is changed. It markedly differs from fresh engine oil as it contains minute quantities of additives and metallic salts. It also contains higher concentrations of heavy metal contaminants that are dangerous to living organisms such as lead, zinc, calcium, barium and magnesium as well as lower concentrations of iron, sodium, copper, aluminum, chromium, manganese, potassium, nickel, and molybdenum resulting from engine wear (Mumford et al. 1986; Vazquez-Duhalt and Greppin 1986).

There is serious environmental concern on the composition of the additives used in engine oil as some of them including zinc diaryl or diakyl dithiophosphates, molybdenum disulphide, heavy metal soaps and organometallic compounds, which contain heavy metals, are dangerous environmental contaminants (Vazquez-Duhalt 1989).

Due to high temperature and mechanical strains the engine oil is subjected to during engine operation, the oil is chemically transformed by oxidation, nitration, cracking of polymers and decomposition of organometallic compounds. Consequent upon this, the waste engine oil accumulates different contaminants such as fuel (petrol or diesel), water, antifreeze and insoluble particles, which principally originates from atmospheric dust, metals, metal oxides and combustion products (Vazquez-Duhalt 1989).

Aside from these dangerous contaminants, waste engine oil also contains other contaminants such as higher percentages of alkyl benzenes, naphthalenes, methylnaphthalenes, polycyclic aromatic hydrocarbons (PAHs) due to pyrosynthesis and chlorodibenzofurans (Wang et al. 2000; Dominguez-Rosado and Pitchell 2003; Lu and Isaac 2008). The PAHs content of new engine oil is relatively low, but increases with engine operation time (Pruell and Quinn 1988; Wong and Wang 2001). Disposal of waste engine oil that is rich in PAHs is a serious health and environmental concern because some PAHs are known to be mutagenic and carcinogenic (Obayori et al. 2008; Salam et al. 2014).

Generally, waste engine oil enters the environment through accidental spills, indiscriminate disposal and operations by automobile mechanics. The Nigerian environment is characterized by nonchalant, indiscriminate and highly unregulated disposal of petroleum products including engine oil (Odjegba and Sadiq 2000; Obayori et al. 2010). Automobile workshops often dispose waste oil on open grounds where it subsequently finds its way into drainages, canals and underground water resulting in gross pollution and constituting potential threats to humans, animals, soil and vegetation (Edewor et al. 2004; Bagherzadeh-Namazi et al. 2008). Observations had shown that even small release of petroleum hydrocarbons into aquifers could lead to concentrations of dissolved hydrocarbons far in excess of regulatory limits (Spence et al. 2005).

Prolonged exposure and high oil concentration may cause the development of liver or kidney disease, possible damage to the bone marrow and an increased risk of cancer (Lloyd and Cackette 2001; Mishra et al. 2001). They are capable of causing different undesirable changes in the environment and in the anatomical features of man (Akoachere et al. 2008). Hydrocarbon pollution have been reported to have inhibitory effect on photosynthesis of phytoplankton communities, as waste engine oil is one of the most important mutagenic agents in the aquatic environment (Vazquez-Duhalt 1989).

Furthermore, research findings have shown statistically significant impact of such reckless disposal on plants, including height reduction, chlorophyll loss and protein level reduction (Oluwole et al. 2005; Umechuruba 2005).

Bioremediation is the exploitation of degradative competencies of microorganisms to remove the environmental pollutants and recalcitrant xenobiotics (Habe et al. 2001). Bioremediation remains one of the most effective ways to reclaim soils and aquifers polluted with petroleum hydrocarbons. Such efforts would depend on the availability of petrophilic organisms with capacity to degrade the broad array of components in the contaminant. Reports abound of degraders of engine oil and waste engine oil spanning strains of genera such as *Acinetobacter*, *Achromobacter*, *Arthrobacter*, *Flavobacterium,* and *Pseudomonas* among others (Adelowo et al. 2006; Mandri and Lin 2007; Bagherzadeh-Namazi et al. 2008; Basuki et al. 2011; Obayori et al. 2014; Salam et al. 2015).

Oftentimes, individual strains are only capable of degrading a few components of the oil pollutant, and complete biodegradation require the activity of consortium (Lal and Khanna 1996; Adebusoye et al. 2007). However, there is increasing research in the isolation of individual organism that can not only degrade the major components of engine oils but also demonstrate versatility for other more recalcitrant hydrocarbons, as oftentimes these pollutants are found together in the same environmental compartments. Here, we report the degradation of waste engine oil by two *Pseudomonas aeruginosa* strains isolated from tropical hydrocarbon-contaminated soil in Lagos, Nigeria.

## Materials and methods

### Spent engine oil

Waste engine oil of grade SAE 40W was collected from a local automobile workshop. The waste oil was collected in well-washed and air-dried plastic bottles.

### Determination of heavy metals content of waste engine oil

Waste engine oil was ashed at a temperature of 600 °C for 6 h, digested with 10 ml 0.02 M nitric acid and analyzed for heavy metal concentrations using flame atomic absorption spectrophotometer Phoenix-986.

### Sampling

Soil samples for this study were collected from an automobile workshop in Lagos, Nigeria. The coordinates of the sampling site were latitude 6°28′ 20.59″N and longitude 3°21′ 00.48″E, respectively. Soil samples were collected at a depth of 10–12 cm using sterile hand trowel after clearing debris from the soil surface. Samples for microbiological analysis were collected in sterile screw-capped bottles. Immediate analysis of the samples were carried out within 5 h of collection or stored at 4 °C.

### Enrichment and isolation of waste engine oil degrading bacteria

Bacteria able to degrade waste engine oil were isolated on carbon free mineral salts medium (CFMM) amended with waste engine oil by continual enrichment method. The carbon free mineral medium (CFMM) described by (Habe et al. 2002) was used. The medium contained per liter of distilled water NH_4_NO_3_, 3.0 g; Na_2_HPO_4_, 2.2 g; KH_2_PO_4_, 0.8 g; MgSO_4_.7H_2_O, 0.1 g; FeCl_3_.6H_2_O, 0.05 g; and CaCl_2_.2H_2_O, 0.05 g. The medium was supplemented with yeast extract (0.005 g). The pH of the medium was adjusted to 7.0 and nystatin included at 50 µg/ml to arrest fungal growth.

Contaminated soil (5 g) was added to 45 ml of CFMM containing 1 ml of spent engine oil. Enrichment was carried out by incubation with shaking (180 rpm) at room temperature (29 ± 2 °C) in the dark for 2–3 weeks until there was turbidity. After four consecutive transfers, waste engine oil degraders were isolated by plating out dilutions from the final flasks on Luria–Bertani (LB) agar. The colonies that appeared were further purified by subculturing once onto LB agar. Ability to degrade waste engine oil was confirmed by inoculating washed LB broth grown culture in fresh CFMM flask supplemented with 1 ml (2 %) waste engine oil as sole carbon source. Two isolates designated strains RM1 and SK1 out of the eight screened, were selected for further study based on its extensive degradative ability.

### Maintenance, identification and characterization of isolates

The pure waste engine oil degrading isolates were maintained in glycerol/LB broth medium (1:1, v/v). Pure colonies subcultured on LB agar supplemented with low percentage of waste engine oil (0.005 %) were harvested with sterile inoculating loop, pooled and transferred to the medium. The mixture was shaken to homogenize and kept at −20 °C.

Identification of the pure waste engine oil degrading isolates was carried out based on their colonial morphology, cellular morphology and biochemical characteristics according to the identification scheme of Bergey’s Manual of Determinative Bacteriology (Holt et al. 1994). This was complemented with API 20 NE V6.0 rapid test kit phenotypic typing (BioMerieux, Durham, NC, USA) according to manufacturer’s instruction. This test kit is a rapid identification system for non-fastidious, non-enteric Gram-negative rod-shaped bacteria.

PCR amplification of the 16S rRNA gene from genomic DNA of strains RM1 and SK1 was performed using the primers 27f (5′-AGAGTTTGATC{A/C}TGGCTCAG-3′) and 1378r (5′-CGGTGTGTACAAGGCCCGGGAACG-3′) (Heuer et al. 1997). The reaction mix contained 20 pmol each of universal primers, 10 µl of Ex *Taq* buffer (Mg^2+^ plus), 2.5 mM of each dNTPs, 2.5 U (0.5 µl) of Ex *Taq* polymerase (Takara) and 1.0 µl of purified genomic DNA in a total volume of 100 μl. Amplification conditions, purification of PCR products and sequencing was done as described previously (Salam et al. 2014). The 16S rRNA nucleotide sequence obtained from both strands was aligned (CLUSTAL W) and the homology search for 16S rRNA was performed in the DDBJ/EMBL/GenBank database using the basic local alignment search tool (BLAST) program. Strains RM1 and SK1 sequences have been deposited in the DDBJ/EMBL/GenBank database under the accession number KU508627 and KU508628.

### Metal tolerance assay

Strains RM1 and SK1 were grown in LB broth for 18 h at room temperature. Cells were harvested by centrifugation (7000×*g*; 10 min), washed twice with sterile phosphate buffer, and resuspended in the same buffer solution. The cell concentration of bacterial suspensions was determined by measuring the optical density of the samples at 600 nm and relating the value to a calibration curve (10^10^ cfu l^−1^ = 1 OD unit).

Stock solutions (1 M) of metal salts namely, NiSO_4_, Pb(NO_3_)_2_, and Zn(NO_3_)_2_ were prepared in distilled water, filter sterilized using 0.22-μm membrane filters, and stored in sterile bottles in the dark at 4 °C. Dilutions to 1, 5, 10, and 15 mM of Zn^2+^, Ni^2+^, and Pb^2+^ were made from the stock solutions into LB broth. The media were dispensed in 5-ml aliquots and inoculated with 50 μl (1 %, v/v) inoculum. Each of the experiment was conducted in triplicates. LB broth not supplemented with heavy metals and inocula serves as controls. Growth of the inocula was measured by absorbance at 600 nm and occasional viable count assay. Resistance was assayed by determining the maximum tolerance concentrations (MTCs) for the isolates after 7 days of incubation. MTC is defined as the highest concentration of metal, which do not affect the viable counts of organisms.

### Evaluation of spent engine oil biodegradation

Waste engine oil degradation potentials of the pure isolates was assayed by inoculating 250-ml replicate flasks containing 50 ml of CFMM supplemented with 1 ml (2 %, v/v) waste engine oil as sole carbon and energy source, respectively. Flasks were inoculated with 0.5 ml of CFMM-washed 18–24 h LB agar-grown cells and subsequently incubated at 180 rpm in the dark for 21 days at room temperature. Flask containing heat-killed cells sterilized at 121 °C for 15 min and supplemented with waste engine oil as described above were used as controls. Samples were withdrawn from each flask at 3 days interval and aliquots of appropriate dilutions were plated (in triplicates) onto nutrient agar for total viable counts (TVC).

### Extraction of residual spent engine oil

Residual waste engine oil was extracted by liquid–liquid extraction. Briefly, broth culture (50 ml) was extracted twice with an equal volume of hexane. After removing the aqueous phase with separating funnel, the organic fraction was concentrated to 1 ml, and the residual concentration of waste engine oil was determined by gas chromatography. Similarly, control flasks were also extracted.

### Analytical method

Hexane extracts (1.0 µl) of residual waste engine oil were analyzed with Hewlett Packard 5890 Series II gas chromatograph equipped with flame ionization detector (FID) and 30 m long HP-5 column (internal diameter, 0.25 mm; film thickness, 0.25 µm). The carrier gas was nitrogen. The injector and detector temperatures were maintained at 250 and 350 °C, respectively. The column temperature was programmed from 60 to 500 °C for 27 min. The gas chromatograph column was programmed at an initial oven temperature of 70 °C; this was held for 2 min, and then ramped at 10 °C/min to 320 °C and held for 10 min.

### Statistical analysis

Mean generation times (*T*_d_) and specific growth rates (*µ*) of the isolate on waste engine oil was calculated using non-linear regression of growth curves for the period when growth rates were maximal using Prism version 5.0 (Graphpad software, San Diego, CA, USA).

## Results

### Heavy metals content of waste engine oil

Spectrophotometric analysis of the waste engine oil used in this study revealed the presence of various heavy metals such as lead, chromium, zinc, copper, nickel, manganese and iron, respectively (Table 1).

**Table 1:** Heavy metal content of waste engine oil

Heavy metal	Concentration (mg/l)
Chromium	0.51
Lead	3.21
Zinc	62.46
Iron	11.03
Copper	0.21
Manganese	0.46
Nickel	25.10

### Isolation, identification and characterization of waste engine oil degrading isolate

Continuous enrichment resulted in the isolation of several waste engine oil degraders. Two isolates with the most extensive degradation ability on waste engine oil were selected for further study. The two isolates were gram-negative, aerobic, non-endospore forming motile rods, catalase and oxidase positive and failed to ferment all the sugars tested with the exception of d-glucose and d-mannitol. The two isolates utilize l-arginine, capric acid, malic acid, citrate, reduce nitrate to nitrogen gas, liquefies gelatin, grew at 42 °C and are indole negative. Strain RM1 produces urease and β-galactosidase enzymes, assimilate esculin, *N*-acetyl glucosamine, gluconic acid, adipic acid and phenylacetic acid and is putatively identified as *Pseudomonas aeruginosa* strain RM1 (99.9 % similarity). In contrast, strain SKI is urease and β-galactosidase negative and failed to assimilate esculin, *N*-acetyl glucosamine, gluconic acid, adipic acid and phenylacetic acid, and is putatively identified as *Pseudomonas aeruginosa* strain SK1 (87.8 % similarity).

However, molecular characterization of the two strains based on sequencing of 16S rRNA partial fragments (1388 bp) indicates significant alignments of strains RM1 and SK1 with nucleotide sequences of *Pseudomonas aeruginosa* strains deposited in the DDBJ/EMBL/GenBank databases exhibiting 100 % homology.

### Metal tolerance of waste engine oil degrading isolates

Metal tolerance assay of strains RM1 and SK1 on various heavy metals were conducted to determine the tolerance limit of the isolates to various concentrations of heavy metals. The assay revealed different resistance patterns. All the isolates (RM1 and SK1) showed resistance to 1–15 mM zinc and resisted 1–5 mM of nickel and lead, respectively. Lead concentration of up to 10 mM was tolerated by SK1, while strain RM1 tolerated up to 10 mM nickel, respectively.

### Growth kinetics of isolates on waste engine oil

**Fig. 1:**
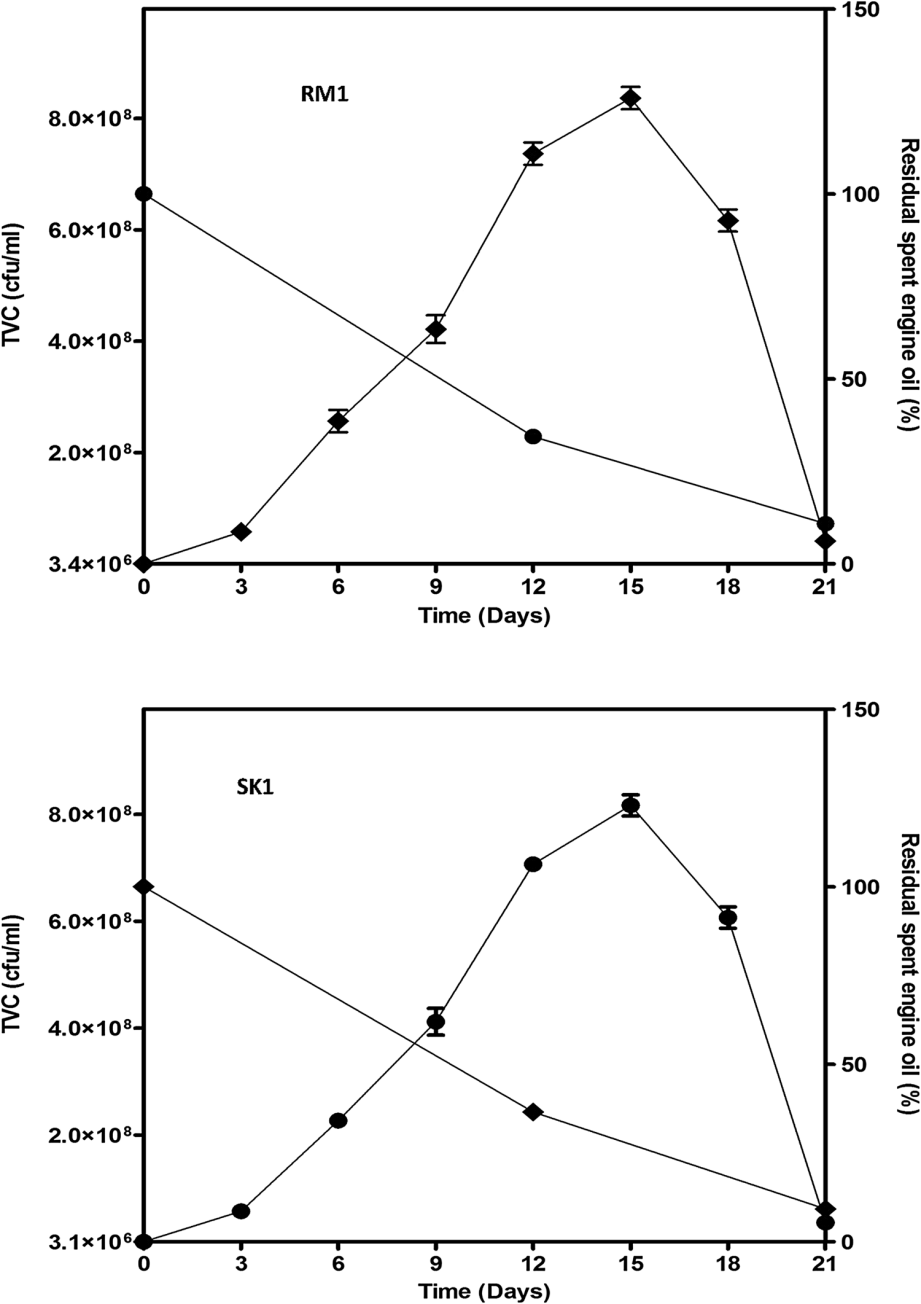
Growth dynamics of *Pseudomonas species* in CFMM amended with 2 % (1 ml) spent engine oil. Spent engine oil was not degraded in flasks inoculate with heat-killed cells. *Data points* represent the mean of three replicate flasks. In the case of population counts, *error bars* represent standard deviation. Residual spent engine oil was determined with reference to spent engine oil recovered from heat-killed controls

Sequel to a 21-day incubation of the isolates on waste engine oil as sole carbon and energy source, significant increase in cell densities was observed with concomitant decrease in different components of the waste engine oil. The growth kinetics of strain RM1 on waste engine oil is depicted in Fig. 1 and Table 2. Strain RM1 grew from an initial population density of 3.40 × 10^6^ cfu/ml to peak at 8.40 × 10^8^ cfu/ml in 15 days. It, thereafter, declined steeply to 4.45 × 10^7^ on day 21. On waste engine oil, the isolate maintained a specific growth rate and doubling time of 0.377 day^−1^ and 1.83 day, respectively. Strain SK1 also grew from an initial population density of 3.10 × 10^6^ cfu/ml to plateau at 8.20 × 10^8^ cfu/ml in 15 days, and thereafter, declined gradually to 3.9 × 10^7^ on day 21. The isolate also maintained a specific growth rate and doubling time of 0.381 day^−1^ and 1.82 day, respectively (Fig. 1; Table 2).

**Table 2:** Growth kinetics of *P. aeruginosa* strains on spent engine oil

Isolate	Growth rate, µ (day^−1^)	Mean generation time, Δ*T*_d_ (d)	PD12 (%)	PD21 (%)	DR12 (mg l^−1^ day^−1^)	DR21 (mg l^−1^ day^−1^)
RM1	0.377	1.83	65.58	89.06	66.30	31.6
SK1	0.381	1.82	63.40	90.75	69.60	40

*PD12* percentage of used engine oil degraded in the first 12 days, *PD21* overall percentage of oil degraded during 21 days incubation, *DR12* degradation rate between Day 0 and Day 12, *DR21* degradation rate between day 12 and day 21

Waste engine oil degradation by *P. aeruginosa* strain RM1 and SK1 was monitored at 72-h intervals in CFMM containing waste engine oil. After 12 days of incubation of strain RM1, the residual waste engine oil content (1212.44 mg/l; 100 %) decreased to 34.42 % (417.33 mg/l) corresponding to removal of 65.58 % (795.11 mg/l) waste engine oil. At the end of 21 days incubation period, the residual waste engine oil decreased further to 10.93 % (132.63 mg/l) corresponding to removal of 89.06 % (1079.81 mg/l) waste engine oil (Fig. 1). Similarly, after 12 days of incubation of strain SK1, the residual waste engine oil content (1317.31 mg/l; 100 %) decreased to 36.60 % (482.22 mg/l) corresponding to removal of 63.40 % (835.09 mg/l) waste engine oil. At the end of 21 days incubation period, the residual waste engine oil decreased further to 9.25 % (121.81 mg/l) corresponding to removal of 90.75 % (1195.5 mg/l) waste engine oil. In the heat-killed control flasks, no apparent decrease of the substrate (waste engine oil) was observed (Fig. 1).

### Kinetics of hydrocarbon degradation

**Fig. 2:**
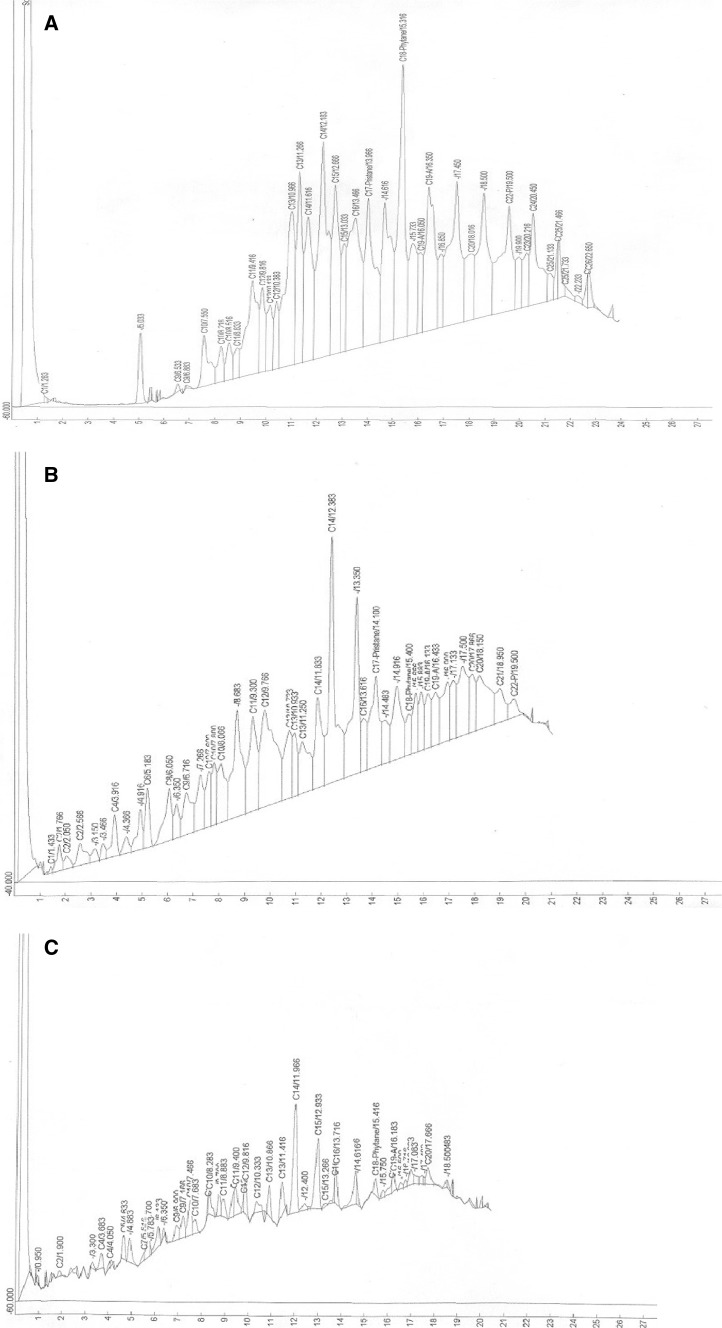
Gas chromatographic traces of *n*-hexane extract of recovered spent SAE 40 engine oil from culture fluids of *Pseudomonas aeruginosa* strain RM1 at Day 0 (**a**), Day 12 (**b**) and Day 21 (**c**) of incubation at room temperature. The oil components were separated on 30 m long HP-5 column (internal diameter 0.25 mm; film thickness 0.25 µm) in a Hewlett Packard 5890 Series II gas chromatograph equipped with flame ionization detector (FID)

**Fig. 3:**
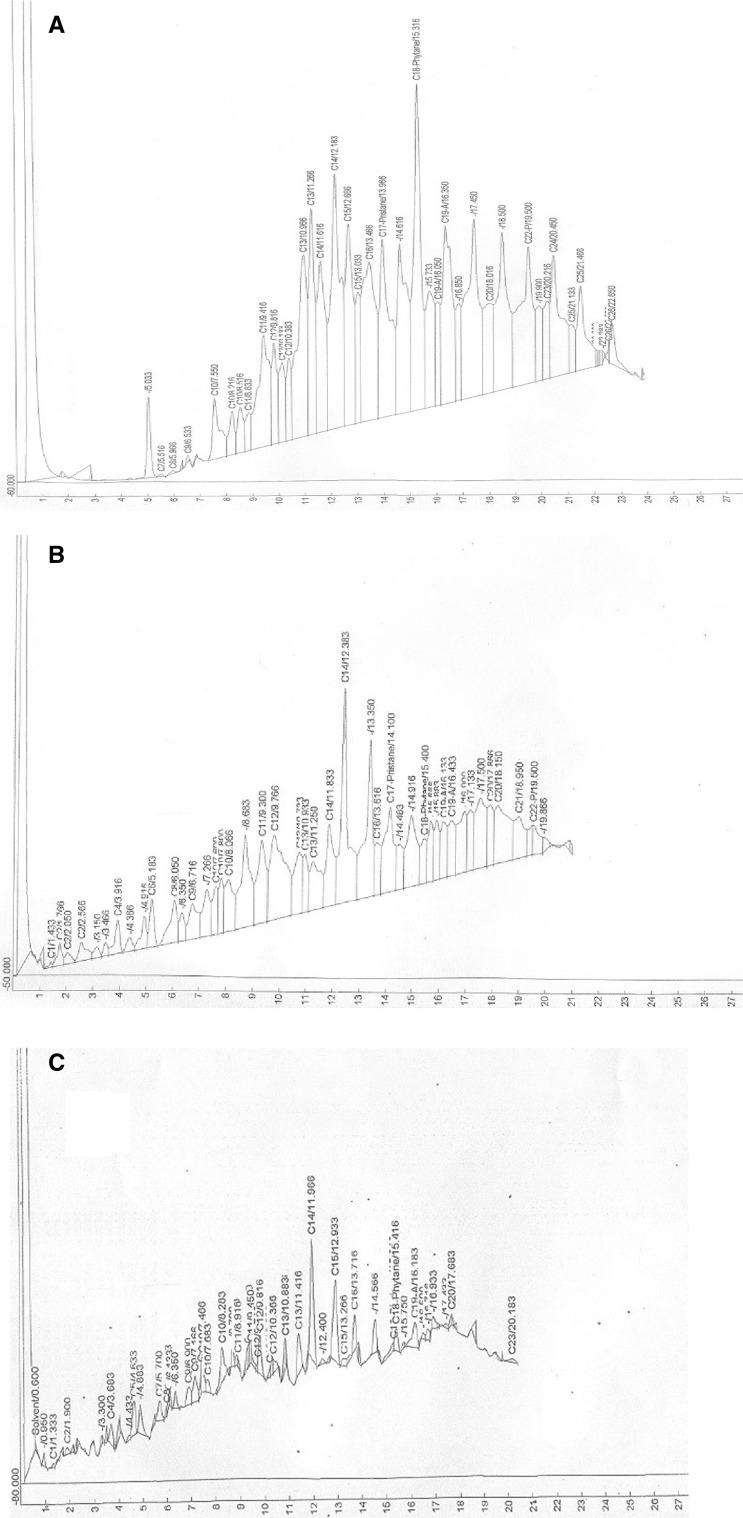
Gas chromatographic traces of *n*-hexane extract of recovered spent SAE 40 engine oil from culture fluids of *Pseudomonas aeruginosa* strain SK1 at Day 0 (**a**), Day 12 (**b**) and Day 21 (**c**) of incubation at room temperature. The oil components were separated on 30 m long HP-5 column (internal diameter 0.25 mm; film thickness 0.25 µm) in a Hewlett Packard 5890 Series II gas chromatograph equipped with flame ionization detector (FID)

Significant changes were observed in various hydrocarbon components of the waste engine oil during the degradation process as shown in the GC fingerprints in Figs. 2 and 3. At Day 0, the GC fingerprints of the waste engine oil showed range of hydrocarbon fractions C_9_–C_26_, with C_14_, the highest peak (Figs. 2a, 3a). In the chromatogram of the two isolates, there was a drastic reduction of most of the peaks by day 12, with total disappearance of C_15_ (pentadecane), C_23_ (tricosane), C_24_ (tetracosane), C_25_ (pentacosane) and C_26_ (hexacosane) fractions, respectively (Figs. 2b, 3b). In addition, there was marked reduction of C_13_ (tridecane 79.8 %, strain RM1; 77.5 % strain SK1), C_14_ (tetradecane 58.7 %, strain RM1; 54.5 %, strain SK1), C_16_ (hexadecane 89.8 %, strain RM1; 88.5 %, strain SK1) and PAHs fractions such as C_19_-anthracene (76.8 %, strain RM1; 73.3 %, strain SK1) and C_22_-pyrene (92.9 %, strain RM1; 85.2 %, strain SK1). Interestingly, molecular biomarkers, C_17_-pristane (67.2 %, strain RM1; 64.3 % strain SK1) and C_18_-phytane (92.9 %, strain RM1; 91.2 % strain SK1) were also reduced significantly. Furthermore, lower molecular weight carbon fractions (C_1_, C_2_, C_4_, C_6_, and C_8_) hitherto not present on Day 0 appeared on Day 12 (Figs. 2b, 3b).

In the chromatogram of the two isolates, at the end of 21 days of incubation period, there is a complete disappearance of C_17_-pristane, one C_19-A_ (in strain SK1 only), C_21_, C_22_-pyrene, reappearance of C_15_ and C_23_ (C_23_ in strain SK1 only) and duplicity of peaks of various carbon fractions such as C_11_, C_12_, and C_13_, respectively. Additionally, >90 % of C_13_ (92.7 %, strain RM1; 94.9 %, strain SK1), C_16_ (98.3 %, strain RM1; 96.0 %, strain SK1) and C_18_-phytane (97.2 %, strain RM1; 95.1 %, strain SK1) and >80 % C_14_ (87.3 %, strain RM1; 89.9 %, strain SK1) fractions were removed (Figs. 2c, 3c).

## Discussion

Biodegradation is a process by which microorganisms transform or mineralize the molecular structure of an environmental pollutant through metabolic or enzymatic processes into less harmful, non-hazardous substances, which are subsequently integrated into natural biogeochemical cycles. The mechanisms of adaptation of autochthonous microorganisms to hydrocarbon perturbations includes synthesis of inducible enzymes, mutations such as single nucleotide change or DNA rearrangement that results in degradation of the compound and acquisition of genetic information from closely related or phylogenetically distinct population within the hydrocarbon-challenged community through horizontal gene transfer (HGT) (Top and Springael 2003; Salam et al. 2011).

The present study investigated the degradative ability of two *P. aeruginosa* strains on waste engine oil. The genus *Pseudomonas* encompasses arguably the most diverse and ecologically significant group of bacteria due to their remarkable degree of physiological and genetic adaptability. *Pseudomonas* is reputed to possess broad substrate affinity for different classes of hydrocarbons such as alicyclics, heterocyclics, and aromatics (Vankateswaran et al. 1995; Nojiri et al. 1999; Obayori et al. 2008; Salam et al. 2014).

In this study, the isolates showed extensive degradative abilities on different hydrocarbon fractions of the spent SAE40 engine oil concomitant with increase in population density. The growth rates of strains RM1 and SK1 on waste SAE40 were 0.377 and 0.381 day^−1^, respectively. These values are higher than 0.13 and 0.1 day^−1^ reported for waste engine oil degrading *Pseudomonas* sp. LP5 and *Methylobacterium mesophilicum* strain RD1 isolated from hydrocarbon-contaminated soils in Lagos, Nigeria (Obayori et al. 2014; Salam et al. 2015). The high growth rates may be attributed to previous exposure and consequent adaptation of the isolates to the pollutant as routine indiscriminate disposal of waste engine oil is a regular occurrence at automobile workshops.

Despite the presence of recalcitrant hydrocarbon fractions as well as heavy metals and combustion products in waste engine oil (Table 1), the two *P. aeruginosa* strains degraded more than 60 and 80 % of waste SAE40 engine oil in 12 and 21 days. Strain RM1 degraded 65.58 and 89.06 % while strain SK1 degraded 63.40 and 90.75 % of waste engine oil in 12 and 21 days, respectively. These degradation rates are higher than 60, 71 and 84 % in 28 days reported for *Flavobacterium* sp, *P. aeruginosa* and *Acinetobacter calcoaceticum* isolated from contaminated soils in Kwazulu-Natal, South Africa (Mandri and Lin 2007). It is equally higher than 81 % in 28 days and 89.5 % in 21 days (strain SK1 only) reported for *P. aeruginosa* and *Methylobacterium mesophilicum* strain RD1 isolated from hydrocarbon-polluted sites (Thenmozhi et al. 2011; Salam et al. 2015). It is, however, lower than 93 % in 21 days reported for *P. aeruginosa* LP5 (Obayori et al. 2014).

Significant reduction in peaks between Day 0 and Day 12 concomitant with exponential growth of strains RM1 and SK1, which extend to Day 15 indicate that the degradation of the waste engine oil is growth associated with the isolates utilizing the hydrocarbon substrate as carbon and energy source. The total disappearance of C_15_, C_23_, C_24_, C_25_ and C_26_ hydrocarbon fractions on Day 12 suggests that the fractions may be saturated alkanes, which are amenable to complete degradation or disintegration to shorter hydrocarbon fractions. This possibly explains the emergence of <C_9_ hydrocarbon fractions (C_1_, C_2_, C_4_, C_6_, C_8_) on Day 12 hitherto not present on Day 0.

The drastic reduction by the two strains of C_13_ and C_16_ hydrocarbon fractions by >90 % over the 21 days incubation period indicates the catabolic versatility of this strain. Furthermore, the complete degradation of polyaromatic fractions C_19-A_ (anthracene), and C_22-P_ (pyrene) in the used engine oil by Day 21 as indicated in the GC fingerprints suggest that this isolate possess multiple degradative genes with diverse catabolic ability. This rare ability to degrade aliphatic, aromatic and polyaromatic fractions displayed by the isolates debunk the belief that individual organisms could only metabolize limited range of hydrocarbon substrates (Adebusoye et al. 2007).

Acyclic isoprenoid hydrocarbons such as pristane (2, 6, 10, 14-tetramethyl pentadecane) and phytane (2, 6, 10, 14-tetramethyl hexadecane) are highly persistent during the degradation of crude oil and petroleum products (Bartha and Atlas 1977; Atlas 1981). This explains why they are used as internal biomarkers in environmental hydrocarbon analyses. The persistence may be attributed to either the presence of alkyl branches, which hinder the uptake of the hydrocarbons into the cell, or non-susceptibility of the branches to the enzymes of the β-oxidation pathway (Schaeffer et al. 1979). However, few bacteria are known to be able to oxidize these recalcitrant hydrocarbons. For instance, Nakajima et al. (1985) reported a *Rhodococcus* sp. that have the potential to degrade and utilize phytane, norpristane (2, 6, 10-trimethylpentadecane) and farnesane (2, 6, 10-trimethyldodecane) as sole sources of carbon and energy. Also, Silva et al. (2007) reported the biodegradation of phytane by *Mycobacterium ratisbonense* strain SD4 under nitrogen-starved conditions. In this study, two *Pseudomonas aeruginosa* strains RM1 and SK1 degraded C_17_-pristane (67.2 %, strain RM1; 64.3 % strain SK1) and C_18_-phytane (92.9 %, strain RM1; 91.2 % strain SK1) hydrocarbon fractions of the waste engine oil in 12 days. However, after 21 days of incubation, >95 % of C_18_-phytane fraction was degraded (97.2 %, strain RM1; 95.1 %, strain SK1) while there is complete degradation of C_17_-pristane fraction in the hexane extracts of residual waste engine oil of the two strains. This indicates that strains RM1 and SK1 harbor multiple degradative genes with propensity for degradation of aliphatic, aromatic, polyaromatic and acyclic isoprenoid components of the waste engine oil.

## Conclusion

This study has established the biodegradative ability of two *P. aeruginosa* strains RM1 and SK1 on waste engine oil and their propensity to degrade various hydrocarbon fractions of the oil. It also bring to the fore the potentials of these isolates for bioremediation of spent engine oil affected compartments. Further works to determine the optimum environmental conditions favorable for their application in bioremediation will be the focus of our future research.
